# Mechanisms driving variability in the ocean forcing of Pine Island Glacier

**DOI:** 10.1038/ncomms14507

**Published:** 2017-02-17

**Authors:** Benjamin G. M. Webber, Karen J. Heywood, David P. Stevens, Pierre Dutrieux, E. Povl Abrahamsen, Adrian Jenkins, Stanley S. Jacobs, Ho Kyung Ha, Sang Hoon Lee, Tae Wan Kim

**Affiliations:** 1 Centre for Ocean and Atmospheric Sciences, School of Environmental Sciences, University of East Anglia, Norwich NR4 7TJ, UK; 2 Centre for Ocean and Atmospheric Sciences, School of Mathematics, University of East Anglia, Norwich NR4 7TJ, UK; 3 Ocean and Climate Physics, Lamont-Doherty Earth Observatory of Columbia University, 61 Route 9W, Palisades, New York 10964-1000, USA; 4 Polar Science Center, Applied Physics Laboratory, University of Washington, Seattle, Washington 98105-6698, USA; 5 British Antarctic Survey, Natural Environment Research Council, Cambridge CB3 0ET, UK; 6 Department of Ocean Sciences, Inha University, Incheon 22212, South Korea; 7 Korea Polar Research Institute, Incheon 21990, South Korea

## Abstract

Pine Island Glacier (PIG) terminates in a rapidly melting ice shelf, and ocean circulation and temperature are implicated in the retreat and growing contribution to sea level rise of PIG and nearby glaciers. However, the variability of the ocean forcing of PIG has been poorly constrained due to a lack of multi-year observations. Here we show, using a unique record close to the Pine Island Ice Shelf (PIIS), that there is considerable oceanic variability at seasonal and interannual timescales, including a pronounced cold period from October 2011 to May 2013. This variability can be largely explained by two processes: cumulative ocean surface heat fluxes and sea ice formation close to PIIS; and interannual reversals in ocean currents and associated heat transport within Pine Island Bay, driven by a combination of local and remote forcing. Local atmospheric forcing therefore plays an important role in driving oceanic variability close to PIIS.

## Abstract (web summary)

Pine Island Glacier terminates in a rapidly melting ice shelf and ocean conditions are believed to influence its contribution to sea level rise. Here, the authors show that variability in these ocean conditions is driven by a combination of changes in ocean circulation and local surface heat fluxes.

The ice shelves of the Amundsen Sea buttress a large portion of the West Antarctic ice sheet, protecting it from collapse^1^,^2^. The deep ocean temperatures close to the Amundsen Sea continental shelf are some of the warmest around Antarctica and have warmed by approximately 0.2 °C per decade since the early 1990s (refs 3, 4), although data on the Amundsen Sea continental shelf itself are sparse and no significant trend is evident^5^. The glaciers in the Amundsen Sea sector already produce the greatest mass loss contribution of all the major drainage basins in Antarctica, and this mass loss increased by 59% between 1992 and 2006 (refs 6, 7). Several studies have suggested that the irreversible retreat of some of the Amundsen Sea ice stream grounding lines is imminent or already under way^8^,^9^,^10^,^11^, due to a combination of ocean forcing^5^,^12^,^13^,^14^ and backwards-sloping bedrock^9^,^10^,^12^. Therefore, understanding the mechanisms that control ocean conditions within the Amundsen Sea is of paramount importance.

The ocean-driven melting of the Pine Island Ice Shelf (PIIS) arises as a result of the relatively warm, salty circumpolar deep water (CDW) that floods the lower part of the water column across the Amundsen Sea continental shelf. The thermocline between 300 and 700 m separates this CDW from the colder but fresher winter water (WW) layer above^5^,^13^. The water that reaches the grounding line of Pine Island Glacier (PIG) (where the ice shelf goes afloat) must pass between a ridge at around 700 m below sea level and the bottom of the ice shelf at around 350 m below sea level^12^. Therefore, the depth of the thermocline and the associated average temperature between these depths determines the heat available for melting within the cavity beneath PIIS. There is particular sensitivity to the ocean temperature at the grounding line, where the highest melt rates are observed^12^,^15^; the water reaching the grounding line is likely to originate from around 700 m depth as it enters the cavity^5^.

CDW reaches PIIS via a network of glacially carved troughs in the seabed^13^. CDW primarily flows onto the continental shelf through shelf-edge depressions at 113°W and 102–108°W (henceforth the central and eastern troughs, respectively; Fig. 1a; (refs 16, 17, 18, 19)). An undercurrent flowing eastwards along the continental slope^20^ beneath the surface-intensified westward Antarctic Slope Current turns onshore at these locations. Several processes have been suggested to explain the transport of water onshore at the continental shelf break; these include topographic steering, vortex stretching that pushes the current southwards on the downstream side of the depression^21^, and on-shelf flow due to upslope transport in the bottom Ekman layer^22^. The CDW that reaches PIIS appears to be a combination of water originating from both the central and eastern troughs^19^, which join paths and mix before flowing southwards into Pine Island Bay (74–75°S, 105–100°W).

The hydrographic conditions within the Amundsen Sea are known to vary seasonally^17^,^23^ and interannually^5^,^13^,^14^,^24^. Various model studies have found a relationship between wind forcing at the continental shelf edge and variability in the onshore flux of CDW^17^,^18^,^25^, and observational studies provide some support for this hypothesis^5^. However, the processes that modify the ocean advective heat transport between the shelf break and the ice shelf cavities (approximately 450 km apart) are not well understood. Furthermore there is growing evidence that intense surface heat and salt fluxes within coastal polynyas can modify the ocean forcing of PIIS^26^ and similar ice shelves^27^. Understanding of the variability of CDW transport into and within the Amundsen Sea has been limited by lack of observations, especially during winter. Various research cruises have provided snapshots of the hydrographic conditions during summer, but until several mooring recoveries in 2014 only a single 11.5-month near-bottom record had been obtained in Pine Island Bay^28^.

Here we present mooring observations spanning the continental shelf leading to PIIS. These include a 5-year record of temperature and salinity (the BSR5/iSTAR9 time series) located within 10 km of the southern edge of PIIS, where the main meltwater outflow has been observed^5^,^13^,^29^,^30^. We compare this with three other 2-year mooring records on the continental shelf and a combined time series from three consecutive moorings in the central trough at the continental shelf edge. Of these latter moorings, BSR12 (2009–2011) and iSTAR1 (2012–2014) were almost co-located (1.5 km apart and within one Rossby radius) on the continental shelf at approximately 605 m depth, while BSR13 (2011–2012) was 22.3 km away, on the continental slope at approximately 1,075 m depth. We find that there is considerable variability in the ocean conditions close to PIIS, which is not replicated in the shelf-edge moorings. Instead, it seems that local surface fluxes and changes in the ocean circulation within Pine Island Bay drive the changes observed close to the ice shelf.

## Results

### Variability in ocean conditions within Pine Island Bay

**Figure 1:**
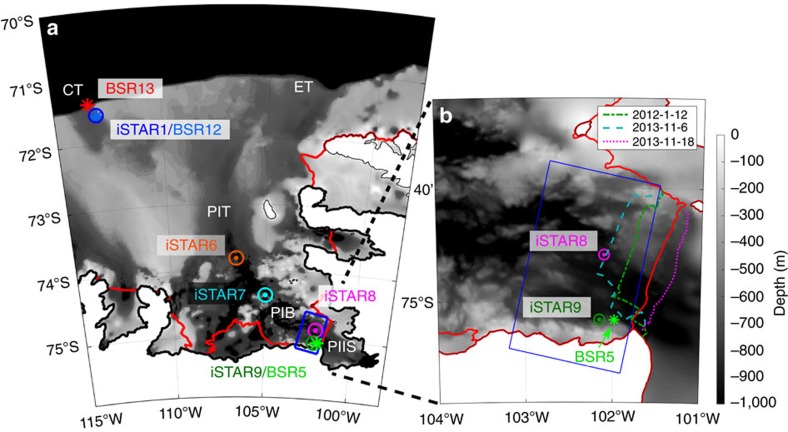
Ocean observations in the Amundsen Sea. (**a**) Location of study area; IBCSO bathymetry^41^ (shaded, see legend) and ice shelf edges from 2004 (red outlines); central (CT), eastern (ET) and Pine Island (PIT) troughs, Pine Island Bay (PIB), Pine Island Ice Shelf (PIIS); mooring locations (labelled; iSTAR moorings circles; BSR moorings stars) and the region over which the surface fluxes are averaged (blue box). (**b**) Enlargement of the Pine Island ice front showing the position of the ice front on 12 January 2012 (green dot-dash line), 6 November 2013 (before iceberg calved; cyan dashed line), 18 November 2013 (after iceberg calved; magenta dotted line).

**Figure 2:**
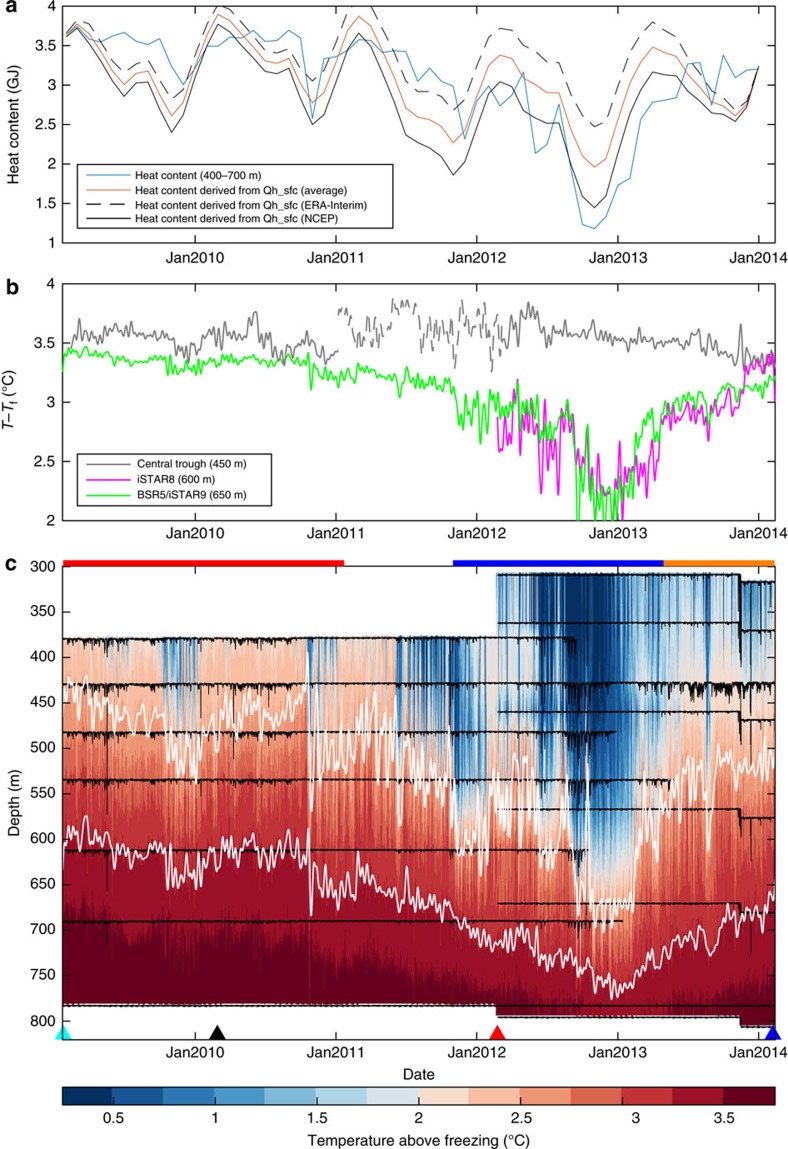
Time series from BSR5 and iSTAR9 compared with surface forcing. (**a**) Monthly heat content between 400 and 700 m on BSR5 (blue) and the heat content derived from the average of NCEP and ERA-Interim monthly mean surface heat flux over the Pine Island Polynya region (red; see methods), with the separate estimate from NCEP (solid) and ERA-Interim (dashed) reanalyses given by the black lines. (**b**) Ten-day low-pass filtered time series of depth-interpolated temperatures above *in situ* freezing point at 450 m on BSR12 (grey, 2009–11), BSR13 (grey dashed, 2011–12) and iSTAR1 (grey, 2012–14); at 600 m on iSTAR8 (magenta) and at 650 m on BSR5/iSTAR9 (green). (**c**) Depth-interpolated temperature above *in situ* freezing point from all available instruments on both moorings, the depth of these instruments is shown by the black lines; the isotherms at 2.5° and 3.25 °C above freezing are shown by the upper and lower white lines, respectively; the timings of the ship-based measurements shown in Fig. 3 are indicated by colour-coded triangles. The warm, cold and subsequent warm periods are indicated by the thick red, blue and orange lines above (**c**).

The time series of temperature above *in situ* freezing point for BSR5 and iSTAR9 (green markers in Fig. 1b) shows considerable variability (Fig. 2c). There is a marked seasonal cycle evident in the depth of the thermocline (for example, the depth of the 2.5 °C above freezing isotherm, upper white line). This seasonal cycle is typified by a deep thermocline and cold temperatures in austral spring (October–December) and generally warmer conditions in all other seasons. Superimposed on this seasonal cycle are interannual changes in temperature, with a pronounced cold period extending from October 2011 to May 2013 (thick blue line above Fig. 2c). During the peak of this cold period, the seasonal cycle is amplified, extending to greater depths and with cold conditions persisting for longer than during the relatively warm period of February 2009 to February 2011 (thick red line above Fig. 2c). The depth of the 3.25 °C above freezing isotherm (lower white line, Fig. 2c) does not have a marked seasonal cycle and is dominated by relatively smooth interannual variability. The temperature minimum at all depths occurs between October 2012 and January 2013, with the deep temperatures lagging behind the shallow temperatures by up to 3 months.

**Figure 3:**
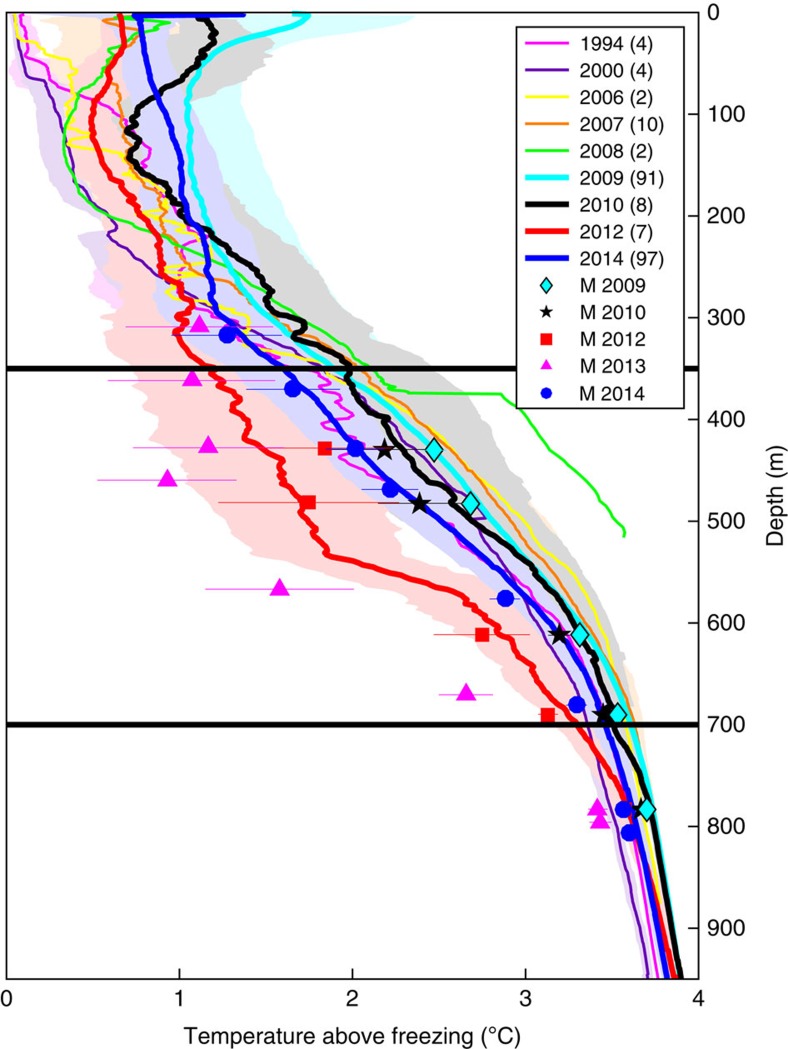
Interannual variability in Pine Island Bay. Summer temperatures close to Pine Island Bay (south of 74°S, east of 105°W) from research cruises (coloured lines, year and number of profiles indicated in legend, 1 s.d. shaded), and mean January–February mooring observations from BSR5 and iSTAR9 (symbols, year indicated in legend, 1 s.d. error bars). Black horizontal lines show the approximate depth of the ice draft and sub-cavity ridge.

We investigate whether the cold conditions observed in 2012–13 were unusual by calculating the average temperatures from all ship-based observations within Pine Island Bay (south of 74°S and east of 105°W), and comparing these with austral summer (January–February) mean temperature data from the BSR5/iSTAR9 time series (Fig. 3). The mooring data are consistent with the ship data for the same years, and it is apparent that temperatures between 350 and 700 m are colder during 2012 than any other summer when ship-based observations exist for this location. The mooring data show that the austral summer of 2013 (shortly after the peak of the cold period) was colder still.

The temperature from shorter mooring records in the region sheds light on the connections between the temperature variability observed at BSR5/iSTAR9 and broader oceanographic changes. The temperature time series at iSTAR8 (Fig. 2b, magenta line) matches very well to that at BSR5/iSTAR9 (Fig. 2b, green line) during its 2-year deployment. Similar variability is also seen at iSTAR7, on the edge of the bay (around 100 km away), and to a lesser extent at iSTAR6, almost 200 km away in Pine Island Trough (Supplementary Fig. 1). Therefore, the variability observed at BSR5/iSTAR9 represents large-scale changes that extend across Pine Island Bay.

At the peak of the cold period in spring 2012, the total ocean heat content above freezing (see Methods) between 400 and 700 m at BSR5/iSTAR9 was 1.25 GJ, a 62% reduction from the 3.28 GJ observed in spring 2009 (Fig. 2a). The reduction in temperature above freezing between spring 2009 and spring 2012 was 71% at 400 m, 70% at 500 m, 50% at 600 m, and 20% at 700 m. The average heat content between 400 and 700 m for the entire cold period was 2.37 GJ, a reduction of 32% relative to the 2009–10 average of 3.49 GJ. Due to the relationship between ocean temperature and buoyancy-driven flow along the ice boundary within the cavity, observed basal melt rates are assumed to respond quadratically to changes in temperature above freezing^31^,^32^, which would amplify the impact of these changes on the melt rate of PIIS. With only a single point measurement of temperature and salinity close to the depth of the meltwater outflow (430 m on BSR5), we cannot quantitatively assess the impact on melt. However, a ship-based survey in early 2012 showed that meltwater production had reduced to less than half of its 2009–10 value^5^, so it is reasonable to assume that the reduction in melt at the peak of the cold period was even greater.

The PIIS front advanced considerably between 2009 and 2013, almost reaching the location of iSTAR8 before a large iceberg calved in November 2013 (see coloured lines in Fig. 1b). This event was responsible for moving iSTAR9 downslope (visible in the time series of the depths of the moored sensors, black lines in Fig. 2c), but otherwise caused no major shift in the temperature (shading in Fig. 2c) or currents (Supplementary Fig. 2) observed at BSR5/iSTAR9. The changing shape of the ice tongue may have influenced the circulation around Pine Island Bay and the distribution of the polynyas. However, it seems unlikely that this alone could account for the deep ocean changes in temperature and velocity that are observed across Pine Island Bay.

### The impact of local surface heat fluxes

One hypothesis for the variability within Pine Island Bay is changes in the heat flux at the ocean surface. Heat loss at the ocean surface will drive progressive cooling and deepening of the mixed layer, potentially explaining the near-freezing temperatures observed at 430 m during the cold period (Fig. 2c; Supplementary Fig. 2). In addition, localized intense heat loss could lead to small-scale deep convective chimneys that then mix laterally to cool the intermediate water masses^33^. To analyse quantitatively the impact of surface cooling we compare the change in heat content above freezing between 400 and 700 m with the total heat flux across the ocean surface due to atmosphere-ocean exchange and due to sea ice production and melt^34^,^35^ averaged over the blue box in Fig. 1 (approximately 101.5–103.2°W, 74.6–75.2°S). All reanalyses in this region are prone to errors and uncertainties^36^, as shown by the differences between the ERA-Interim^37^ and NCEP-38 derived heat fluxes in Fig. 2a; therefore, we use the average of the two products as the best guess. An evaluation of the importance of the additional heat flux derived from sea ice formation and melting is provided in Supplementary Fig. 3.

To compare the surface heat flux with the observed ocean heat content changes, we first remove the time-mean heat flux, assuming that the net heat loss at the surface is balanced by a steady supply of heat by ocean advection and mixing, an assumption that we investigate in the following section. In addition, we ignore the largely unobserved changes in heat content above 400 m; during the cold period the temperature at 400 m is close to the surface freezing point, so we assume that the layer above is isothermal, meaning that the unobserved changes will be negligible. However, when the mixed layer is shallow (during periods of warming or generally warmer conditions), the influence of surface fluxes on the deeper water masses will be less than our calculation suggests.

Despite these simplistic assumptions, the observed changes in heat content agree well (*r*=0.65) with those derived from accumulated surface heat fluxes (Fig. 2a), suggesting that the surface heat fluxes can explain most of the seasonal variability in ocean temperature, and a limited portion of the interannual variability. Between spring 2009 and 2012, the surface heat fluxes can account for a reduction in heat content of 0.7 GJ, one-third of the 2.1 GJ reduction observed (Fig. 2a). The simulated heat content in spring 2009 is lower than observed, probably due to the relatively shallow thermocline leading to heat content changes in the upper water column, at depths shallower than the observations. For similar reasons the warm peaks in 2010 and 2011 are over-estimated. The under-estimation of the cold conditions in 2012 suggests that additional processes, such as changes in ocean heat advection, may also have contributed to the cooling during 2011 and 2012.

### The impact of changes in ocean circulation

**Figure 4:**
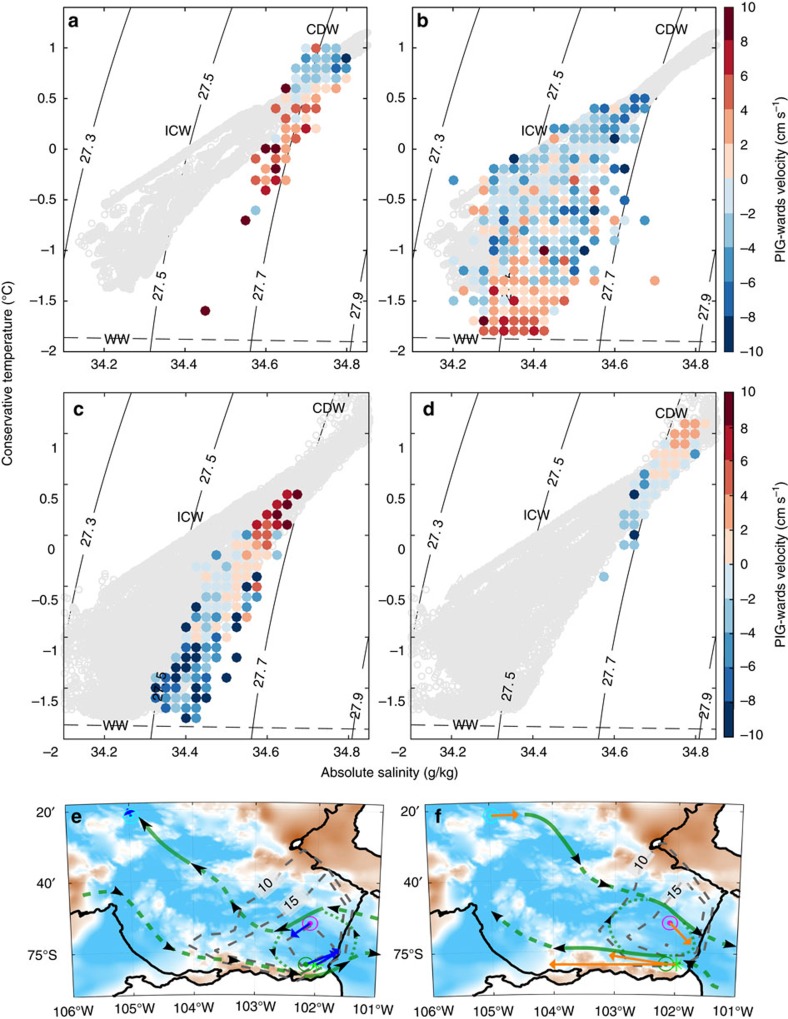
Temperature-salinity diagrams coloured by PIG-wards velocity and oxygen concentration. (**a**) Binned (see methods) T and S at 670 m coloured by corresponding PIG-wards velocity at 676 m (all iSTAR9; 2012–14). (**b**) Binned T and S at 362 m (iSTAR9; 2012–14) and 430 m (BSR5; 2009–14) coloured by PIG-wards velocity at 676 m (iSTAR9; 2012–14) and 534 m (BSR5; 2009–14). (**c**) Binned T and S at 403 m coloured by velocity at 432 m (all iSTAR7; 2012–14). (**d**) Binned T and S at 646 m coloured by velocity at 654 m (all iSTAR8; 2012–14). In (**a**–**d**), *in situ* density anomaly is contoured and ship CTD (conductivity, temperature and depth) observations within Pine Island Bay are shown in grey, and the surface freezing point is shown by the dashed black line. (**e**) IBCSO bathymetry in colour, plus average mooring velocity for the cold period (October 2011–May 2013; blue vectors) for iSTAR7 (cyan circle, 480 m), iSTAR8 (magenta circle, 645 m), iSTAR9 (dark green circle, 674 m) and BSR5 (light green asterisk, 534 m), annual total sea ice production averaged over 2011 and 2012 (dashed contours at 10, 15, 20 m), and a schematic representation of the possible mid-level ocean flow (green arrows, dashed lines represent lower certainty). (**f**), as (**e**) but for the warm period (May 2013–February 2014), with annual total sea ice production averaged over 2009, 2010 and 2013.

The temperature variability at 600 m and deeper is characterized by a steady decline in temperature from mid-2010 to late 2012, followed by warming during 2013 into the start of 2014. This smooth variability suggests relatively little influence from surface heat fluxes locally, apart from during the peak of the cold period when the thermocline deepened to around 700 m. The other major source of variability in ocean temperatures in this region is likely to be from lateral advection or shifts in horizontal temperature gradients associated with fronts. As a proxy for this, the ocean current records at iSTAR7, iSTAR8 and BSR5/iSTAR9 are investigated, and compared with water mass properties (Fig. 4). There is a strong connection between the component of the current heading towards PIIS (PIG-wards currents) and water mass properties, with high temperatures across a range of depths (430–670 m) associated with flow towards the ice shelf at iSTAR7 and iSTAR8 and away from the ice shelf at BSR5/iSTAR9. During the cold period, the flow reversed, with the currents at BSR5/iSTAR9 being towards the ice shelf, and those at iSTAR7 and iSTAR8 being away from the ice shelf. While most ocean modelling studies^14^,^26^ and observational evidence^13^,^29^ suggest that the circulation around Pine Island Bay is cyclonic, with outflow at BSR5/iSTAR9, one modelling study^39^ produced an anticyclonic deep ocean circulation with flow into the cavity close to BSR5/iSTAR9, suggesting that this may be a stable alternative flow regime when the thermocline is deep. A schematic representation of these changes is shown in Fig. 4e,f. Note that the clockwise gyre in Pine Island Bay observed during previous cruises is associated with a doming of isopycnals and isotherms in the centre of Pine Island Bay by 100–200 m^13^,^29^; a reversal in this circulation will have been associated with deepening of these isopycnals by several hundred metres.

Changes in temperature and salinity are positively correlated, such that a drop in temperature corresponds to a decrease in salinity and potential density (Fig. 4a–d, Supplementary Fig. 4). The salinity of the WW increases by around 0.2 g kg^−1^ relative to ship-based summer observations (Fig. 4b,c), changing the mixing line between WW and CDW (Supplementary Fig. 5) such that water at all intermediate depths becomes colder (for example, Fig. 4a). The observed changes in WW properties are likely due to a combination of temporal changes in sea ice formation (Fig. 4e,f) and advection of WW from regions with climatologically higher rates of sea ice production. Therefore, the variability within the thermocline can be seen as a combination of isopycnal displacements (likely driven by changes in circulation and the volume of CDW) and changes to the vertical structure of the water column driven by surface forcing, sea ice formation and subsequent mixing.

The observed changes in circulation patterns could be driven by the changes in surface heat fluxes and sea ice production, which would alter the lateral density gradients in and around Pine Island Bay, and thus alter the currents, or by changes in local wind stress. Local zonal wind stress is weakly correlated (r>0.4) with changes in zonal velocity and temperature at BSR5; this correlation is the strongest observed across the continental shelf at any lag (Supplementary Fig. 6). It is also plausible, as has been suggested by several previous studies^5^,^13^,^16^,^17^, that changes in Pine Island Bay are driven by changes in the CDW flux onto the continental shelf 400 km to the north. The discontinuous mooring time series in the central trough (Fig. 2b, grey lines) show that the temperature of the inflowing CDW was relatively steady over the 5-year period of the moorings, and is not consistent with a major deepening of the thermocline at this inflow location. Even if the temperature remains unchanged, the volume flux of CDW could be altered through changes in on-shelf velocity. Measurements of on-shelf velocity at iSTAR1 and BSR12 offer an incomplete representation of the total on-shelf flux of water, but do show that the on-shelf flow was stronger and more variable during the relatively warm 2009–11 period compared with 2012–14 (Supplementary Fig. 7a). However, the overall agreement between the on-shelf velocity and temperatures in Pine Island Bay is weak (Supplementary Fig. 7b). It is therefore unlikely that variability in conditions in the central trough is the dominant driver of the seasonal or interannual variability that we observe in Pine Island Bay.

The other main pathway for warm CDW onto the continental shelf is the eastern trough. Changes in the zonal wind and associated wind stress curl to the north of the eastern trough have been hypothesized^5^ as a driving mechanism for the cold conditions in 2012. The zonal wind between 68–72°S and 100–115°W was very anomalous in 2011, and did not recover to the 30-year mean until late 2013 (Supplementary Fig. 8), which may have contributed to the anomalous conditions in Pine Island Bay during the cold period. If we assume that the shelf-edge wind stress in this region drives on-shelf CDW flux, then cumulative anomalies in this quantity should be related to variability in CDW volume in Pine Island Bay. The anomalously negative wind stress throughout the period is consistent with the longer-term decline in deep temperatures, although not the recovery to warm conditions in 2013–14 (Supplementary Fig. 8). Nevertheless, a reduction in inflow, such as that hinted at by the shelf-edge moorings (Supplementary Fig. 7), could have enhanced the impact of the surface heat flux variability and promoted the local changes in circulation that are responsible for the shorter-term variability.

## Discussion

**Figure 5:**
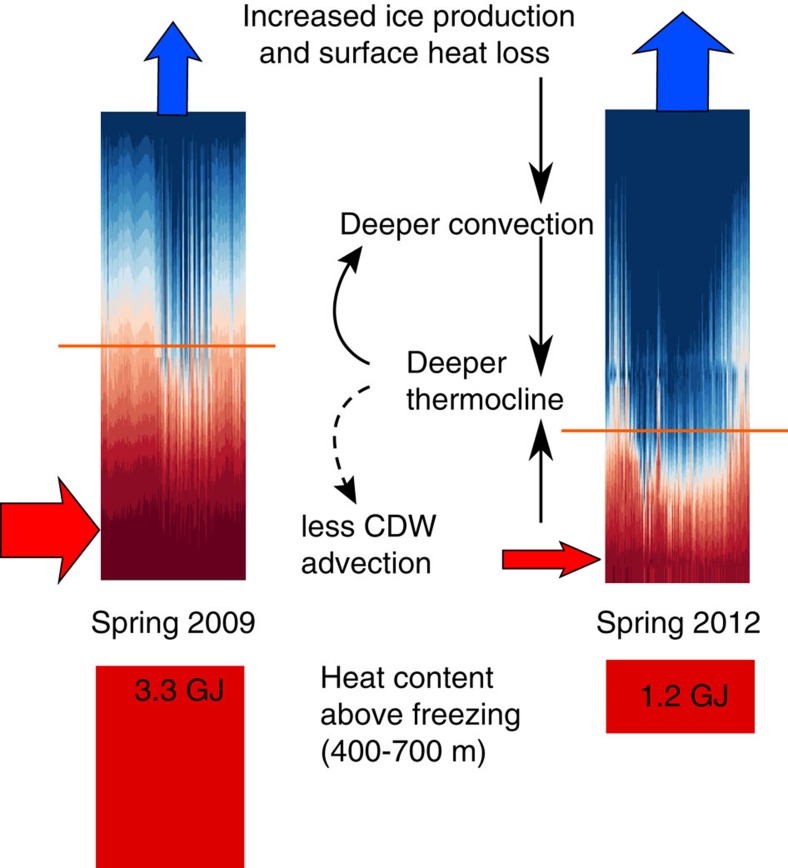
Schematic of heat content variability and proposed mechanism. Temperature profiles and associated heat content above *in situ* freezing point for two representative periods: Spring (October–December) 2009 and 2012; the average heat content above freezing between 400 and 700 m for these periods (red columns and values), and a schematic of proposed mechanisms. Red arrows indicate CDW advection while blue arrows indicated heat loss to the atmosphere. The orange line represents the approximate depth of the thermocline for these periods. Black arrows denote influence from one element to another. The dashed arrow represents the possible influence of a deeper thermocline in Pine Island Bay on the regional circulation and hence CDW advection.

A considerable decrease in temperature above freezing and ocean heat content was observed over a 20-month period that is likely to have had a major impact on melt rates under PIIS that were responsible for a temporary slowing of its outflow^40^. This cold spell seems to arise from a combination of cooling by local surface heat fluxes and changes in circulation and ocean heat advection. Surface forcing within Pine Island Bay can explain much of the observed upper water column (that is, shallower than 600 m) variability close to PIIS at seasonal to interannual time scales, predominantly through cumulative surface heat fluxes (Fig. 5). There is a strong annual cycle in the surface heat fluxes that is also present in the depth of the thermocline, but less evident in the temperature of the water below the thermocline. Nevertheless, through convection and mixing, the surface fluxes can penetrate sufficiently deeply into the ocean to have a major impact on melting close to the grounding line at the peak of the cold spell, and influence the temperature of the water entering the cavity throughout the observed period. The temperature variability in Pine Island Bay is also strongly correlated with changes in circulation, with the cold period of October 2011 to May 2013 associated with a reversal in the currents that transport heat into and around Pine Island Bay. The cause of the circulation change is not known, but it is most likely a combination of local and remote forcing.

We note that a recent modelling study by St-Laurent and others^26^ came to similar conclusions with regard to the importance of sea ice production and heat fluxes in generating a substantial cold event in late 2012. However, their model suggested that this originated in the north of Pine Island Bay during a short period of intense sea ice production and heat loss, while our observational data set suggests that both the heat loss and resultant cooling were more prolonged. In addition, the observed trend in deep ocean temperatures was mostly absent from their model study, as was the reversal in currents. Nevertheless, their model study provides further evidence that local atmospheric conditions can have a substantial impact on intermediate-depth ocean temperatures in this region.

The large interannual variability observed in Pine Island Bay is not clearly linked with changes at the shelf break, although a decrease in the on-shelf currents between the 2009–11 and 2012–14 periods might underline the multi-year decline in deep temperatures. That deep cooling may have enhanced the impact of local processes on the mid-water column, through promoting deeper wintertime convection and changes in circulation within Pine Island Bay. Unfortunately no observations were available within the eastern trough during this period, so we cannot rule out the possibility that changes there drove some of the unexplained changes in heat content in Pine Island Bay. Nevertheless, this study shows that the impact of shelf-edge winds and circulation changes on conditions in Pine Island Bay is less direct than previously suggested and that local atmospheric forcing strongly modulates the response in the critical 350–700-m depth range, at least over the observed period. Therefore, it is likely that other rapidly melting ice shelves across Antarctica will also be strongly influenced by local atmospheric conditions. If confirmed, this would underline the importance of atmospheric and ocean monitoring close to the Antarctic coast to give early warning of future changes in ice shelf melting and glacial retreat.

## Methods

### Rotation of current direction

PIG-wards currents are defined as the maximum in the variance ellipse directed towards PIG. This direction is as follows: iSTAR7, 105°; iSTAR8, 130°; BSR5/iSTAR9, 82°. Figure 4e,f shows that these directions are broadly aligned with the average currents during the warm period. The qualitative findings of this paper are not influenced by the exact angle used to define the PIG-wards direction.

### Binning of temperature and salinity data

Temperature and salinity data are binned for clarity in Fig. 4. Bin edges were defined as every 0.1 °C and 0.025 g kg^−1^, and all data within each bin were given equal weight. The key features evident after binning are also apparent in the raw data, but subsampling or averaging is necessary for these features to be succinctly plotted. Our conclusions are not sensitive to the choice of bin size.

### Temperature above *in situ* freezing point

The temperature above freezing is calculated by subtracting the *in situ* freezing point (calculated using the TEOS-10 equation of state) from the observed temperatures. Since salinity is necessary to calculate the *in situ* freezing point but not observed on the majority of the mooring instruments, a conversion between temperature and salinity is used based upon summer ship observations in the region during 2014. These ship observations show a generally linear relationship (Fig. 4), although there is considerable variability at colder temperatures due to the variation of salinity in the surface layers. The average salinity is calculated in temperature increments of 0.05 °C, and this relationship is then interpolated to the observed temperature. Assuming an uncertainty of ±0.3 g kg^−1^ in salinity, the uncertainty introduced by this conversion is ±0.02 °C for *in situ* freezing point.

### Ocean heat content

Heat content available to melt PIIS is calculated from the temperature above *in situ* freezing point between 400 and 700 m. These depths are used as they lie within the observed range throughout the record, and approximately coincide with the depth range within the cavity beneath PIIS. The heat content is calculated using the following equation:


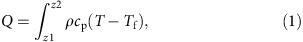


where z1=700 m, z2 is 400 m, *ρ* is the ocean density and *c*_p_ is the ocean heat capacity, both calculated from depth-interpolated temperature (*T*) using the above conversion to estimate salinity where it was not observed. *T*_f_ is the *in situ* freezing point of the water. The uncertainty in density and heat capacity due to unknown variations in salinity is less than 0.1% of their respective average values.

### Data availability

The iSTAR mooring data that support the findings of this study are available from the British Oceanographic Data Centre. The BSR5 mooring data that support the findings of this study are available in the IEDA MGDS repository with the identifier doi: 10.1594/IEDA/322014. The CDT (conductivity, temperature and depth) data that support the findings of this study are freely available in various national data centres; the compiled data set is available from the corresponding author on reasonable request.

## Additional information

**How to cite this article:** Webber, B. G. M. *et al*. Mechanisms driving variability in the ocean forcing of Pine Island Glacier. *Nat. Commun.* **8,** 14507 doi: 10.1038/ncomms14507 (2017).

**Publisher's note**: Springer Nature remains neutral with regard to jurisdictional claims in published maps and institutional affiliations.

## Supplementary Material

Supplementary Information Supplementary Figures.
