# Supplementary material for: Mechanisms driving variability in the ocean forcing of Pine Island Glacier
Source: Nat Commun. 2017 Feb 17;8:14507. doi: 10.1038/ncomms14507 (PMC5321733; doi:10.1038/ncomms14507)
Supplement: Supplementary Information — Supplementary Figures. [file ncomms14507-s1.pdf]

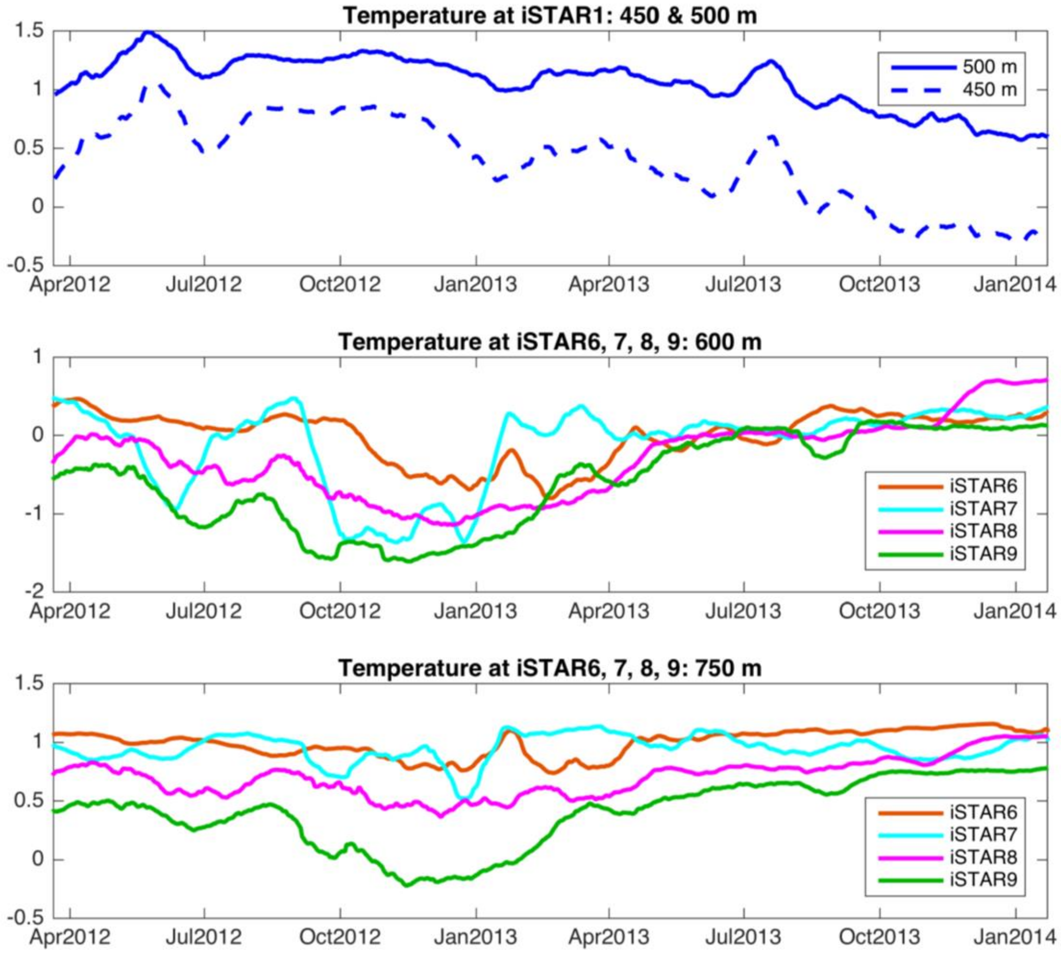

Supplementary Figure 1 | Temperature time series at shelf-break and on-shelf moorings. a, Depth-interpolated in-situ temperature at iSTAR1, at 450 m (dashed) and 500 m (solid). **b,** Depth-interpolated in-situ temperature at 600 m at iSTAR9 (green), iSTAR8 (magenta), iSTAR7 (blue) and iSTAR6 (red). **c,** as **b,** but for 750 m depth. The amplitude of the cold period decreases with distance away from PIIS.

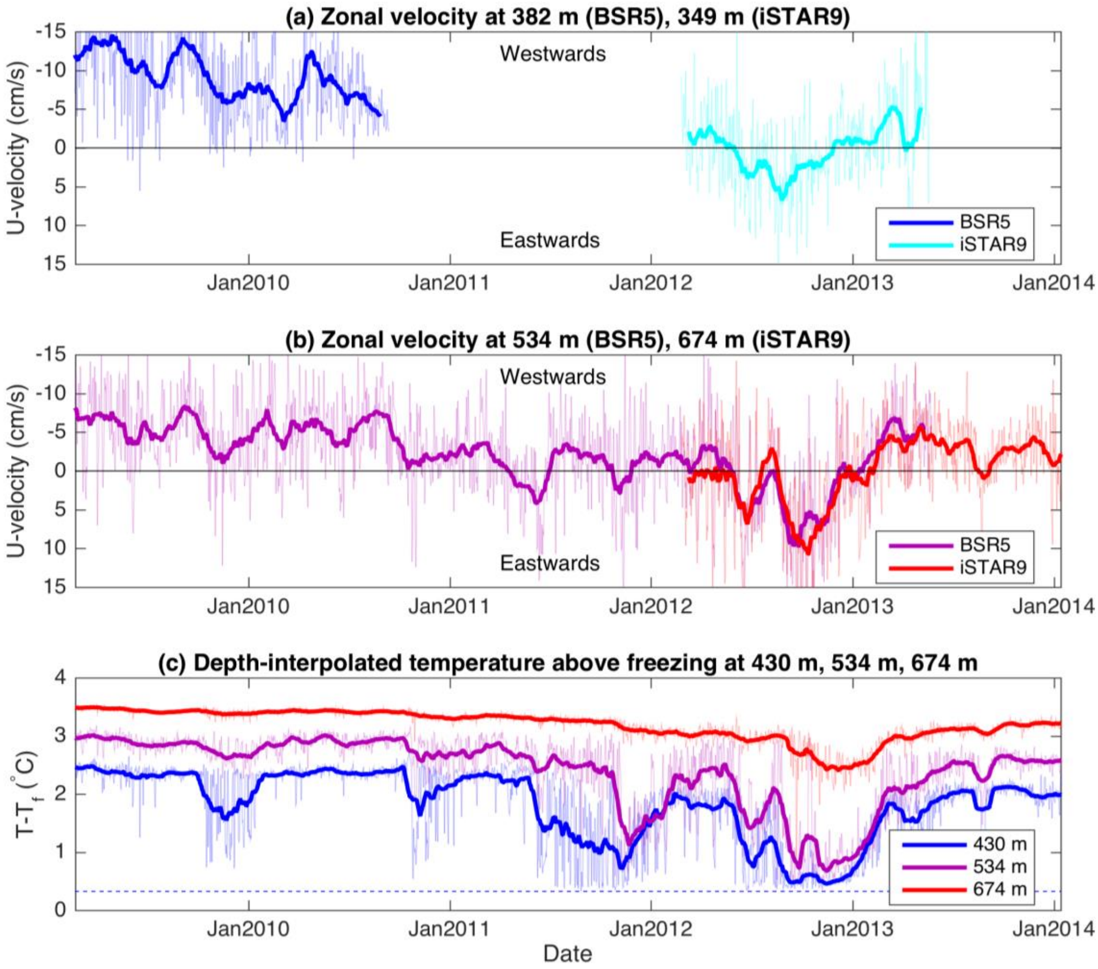

Supplementary Figure 2 | Interpolated temperature and zonal velocities at BSR5 and iSTAR9. a, Zonal velocity at 382 m on BSR5 (blue) and 349 m on iSTAR9 (cyan). **b,** Zonal velocity at 534 m on BSR5 (purple) and 674 m on iSTAR9 (red). **c,** Interpolated temperature above in-situ freezing point at BSR5/iSTAR9: 430 m (blue), 534 m (purple) and 674 m (red). The temperature of water at the surface freezing point above the in-situ freezing point at 430 m is shown by the blue dashed line in **c**. Note the inverted y-axis on **a, b**. Raw data are plotted as faint thin lines; 30-day running means are plotted as thick lines. The correlation between 30-day running means of zonal velocity at BSR5 (534 m) and 30-day running means of temperature above freezing for the overlapping period is: -0.74 at 430 m; -0.71 at 534 m; and -0.64 at 674 m.

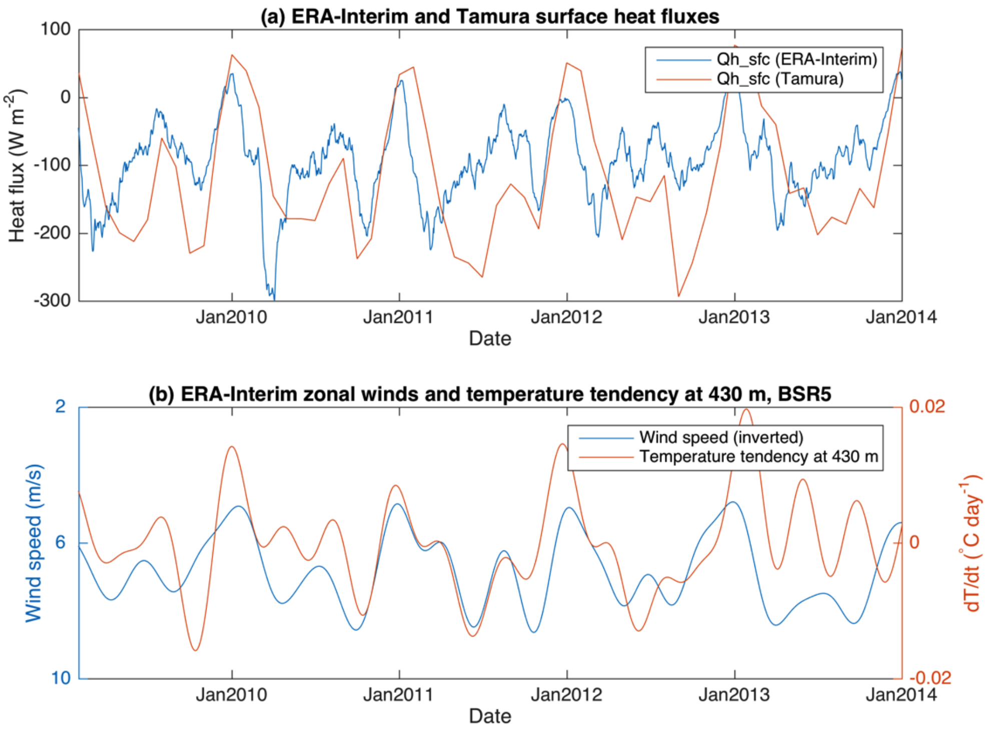

Supplementary Figure 3 | Comparison of heat flux datasets and surface wind speeds. a, Surface heat fluxes over the region 101.8 to 103.2°W, 74.6 to 75.2°S, from the daily ERA-Interim reanalysis[37] (blue line) and the monthly Tamura analysis[35] (red line). **b,** 100-day low-pass filtered wind speed averaged over the region 102 to 110°W, 72 to 75°S (m s^{-1}; blue; note reversed y-axis) and 100-day low-pass filtered temperature tendency from 430 m on BSR5 (°C per day; red).

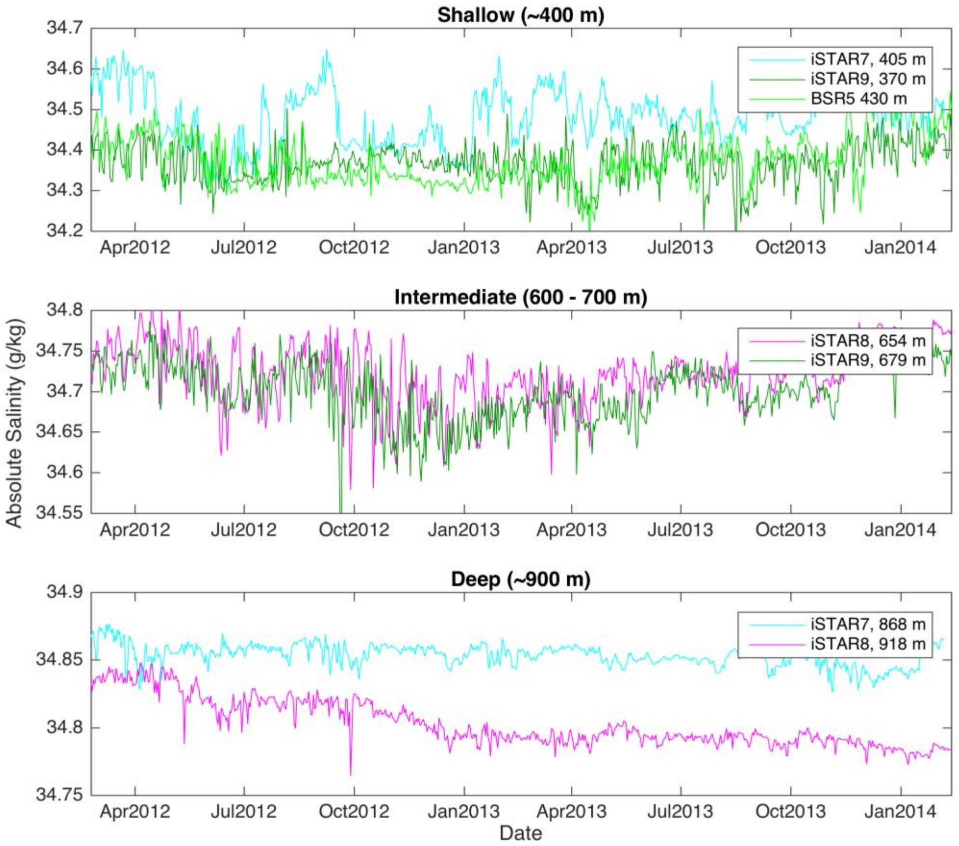

Supplementary Figure 4 | Salinity time series from iSTAR moorings and BSR5. Daily mean salinity data from all salinity observations on iSTAR7 (cyan), iSTAR8 (magenta) and iSTAR9 (dark green), as well as BSR5 (light green). The depths of the observations are shown in the panel legends.

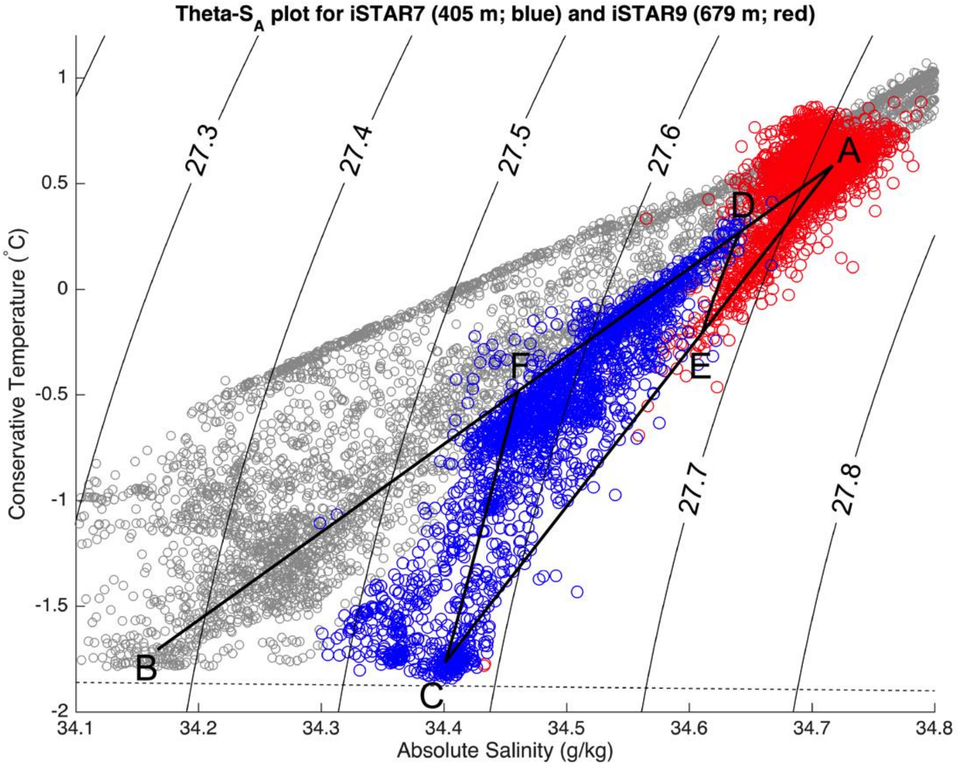

Supplementary Figure 5 | Annotated Theta-S_A plot. Conservative temperature against absolute salinity at iSTAR9 (679 m) and iSTAR7 (405 m). Historical summer CTD observations are shown in grey. The line AB represents mixing between CDW and WW masses across the majority of the region. In contrast, the line AC represents the mixing line between CDW and the higher salinity WW that is observed at iSTAR7 during the cold period. Much of the variability recorded at 679 m at iSTAR9 lies along mixing line AC, suggesting the influence of higher salinity WW extends to this depth. By comparing points D and E, which have the same density but lie on different mixing lines, the temperature change due to vertical movement of isopycnals alone (AD) is 0.5 °C, while the temperature difference including the change in WW properties (AE) is 1.1 °C. The thin solid and dotted lines represent isopycnals (sigma-t) and the surface freezing point, respectively.

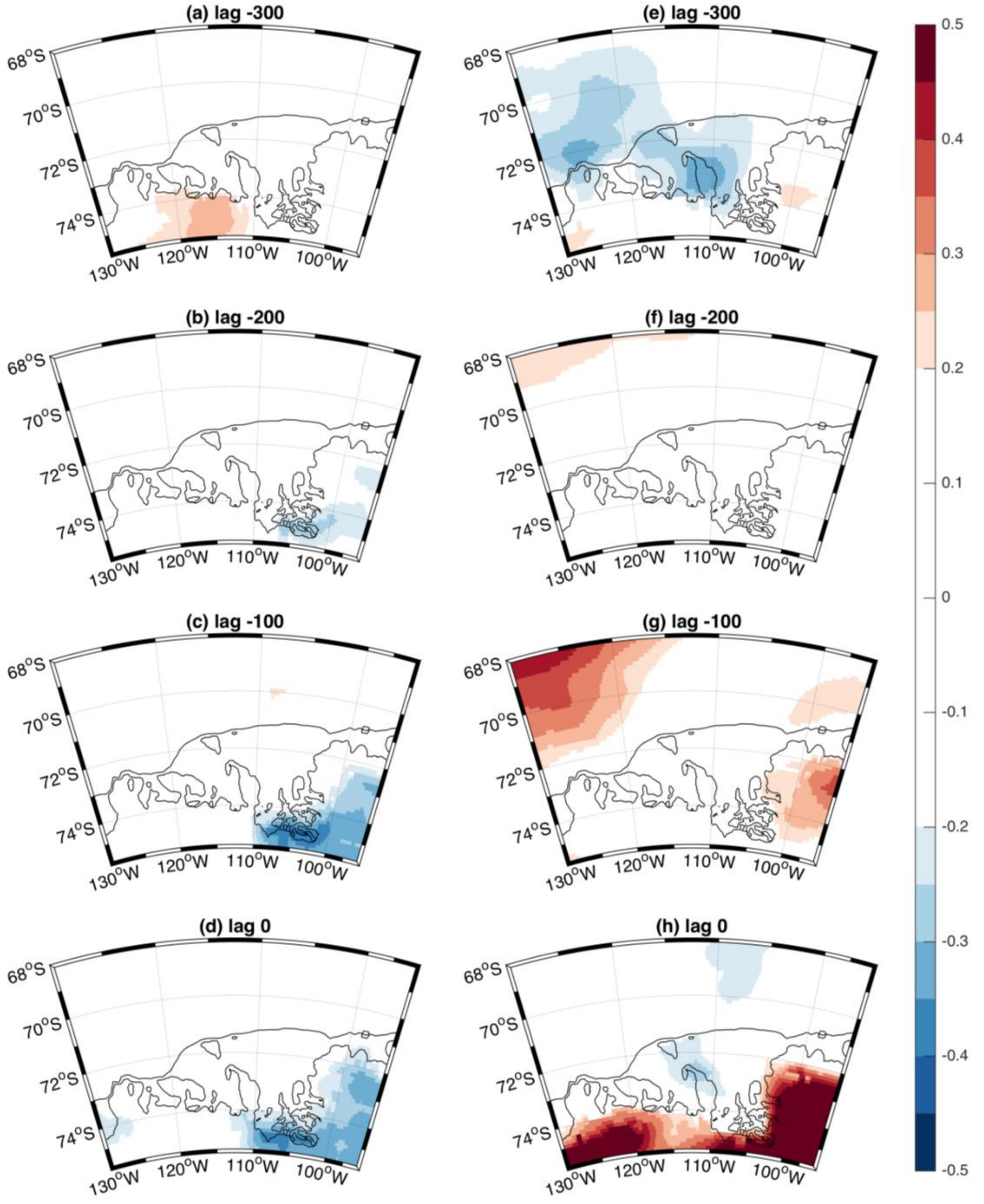

Supplementary Figure 6 | Correlations of zonal wind stress with temperatures and zonal currents at BSR5. a-d, Lagged correlations between 100-day low-pass filtered zonal wind stress and 100-day low-pass filtered temperature at 782 m on BSR5. **e-h,** As for **a-d** but for zonal velocity at 534 m on BSR5. Winds lead ocean parameters for negative lags. Contour lines show IBCSO bathymetry at 500 and 1000 m depth.

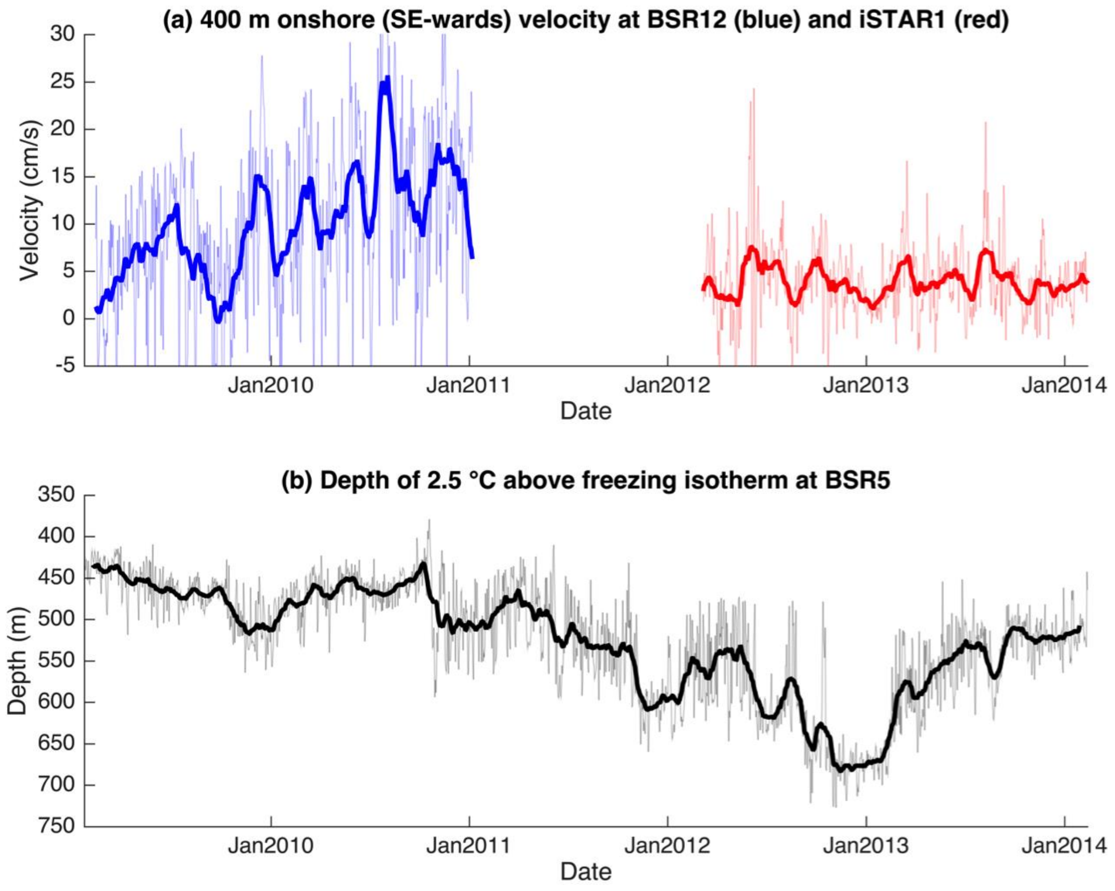

Supplementary Figure 7 | Shelf-edge velocity and thermocline depth at BSR5. **a,** daily mean (faint) and 30-day running mean (bold) velocities at 400 m on BSR12 (blue) and iSTAR1 (red). **b,** daily mean (faint) and 30-day running mean (bold) depth of the 2.5 °C above in-situ freezing point isotherm on BSR5. Although the mean on-shore current speed for the 2012-2014 period is lower than that for 2009-2011, the agreement between the time series is weak. Note that the dynamics of the shelf edge currents makes direct comparison with the intervening BSR13 record impractical.

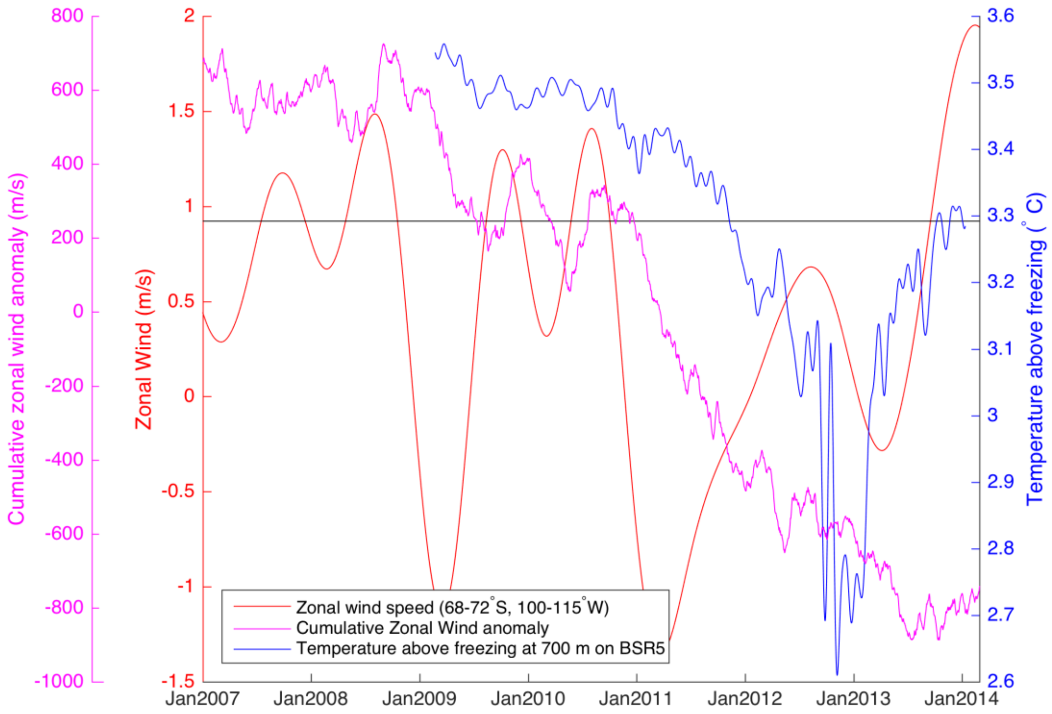

Supplementary Figure 8 | Shelf-break zonal wind and temperature in Pine Island Bay. 365-day low-pass filtered zonal winds averaged over 68-72°S, 100-115°W (red); cumulative zonal wind anomaly (magenta), where the anomaly is calculated by removal of the 30-year mean (black line); 30-day low-pas filtered temperature above freezing at 700 m on BSR5 (blue).
